# Optimization of Cinnamon (*Cinnamomum zeylanicum* Blume) Essential Oil Extraction: Evaluation of Antioxidant and Antiproliferative Effects

**DOI:** 10.1155/2019/6498347

**Published:** 2019-12-24

**Authors:** Imen Kallel, Bilel Hadrich, Bochra Gargouri, Amina Chaabane, Saloua Lassoued, Radhouane Gdoura, Ahmed Bayoudh, Ezeddine Ben Messaoud

**Affiliations:** ^1^Laboratoire de Recherche Toxicologie Microbiologie Environnementale et Santé (LR17ES06), Faculté des Sciences de Sfax, P.O. Box 1171, 3000 Sfax, Université de Sfax, Tunisia; ^2^Unité de Biotechnologie des Algues, Biological Engineering Department, National School of Engineers of Sfax, University of Sfax, Sfax 3038, Tunisia; ^3^Unité de Recherche Biotechnologie et Pathologies, Institut Supérieur de Biotechnologie de Sfax, University of Sfax, Sfax, Tunisia; ^4^Laboratory of Enzyme Engineering and Microbiology, Engineering National School of Sfax (ENIS), University of Sfax, Sfax, Tunisia

## Abstract

Having high cytotoxicity cell line effect, *Cinnamomum zeylanicum* Blume essential oil offers a novel approach to the chemotherapy treatment. In order to enhance its quantity/purity, the experimental conditions to produce essential oil should be more exploited. Steam distillation was used to isolate essential oil, and its conditions' optimization was carried out with the surface-response methodology. The maximum amount (2.6 g/100 g d.b.) was obtained under minimum condensation water flow (0.8 mL/min), a sample size of 6.5 cm, a saline solution concentration of 262.5 g/L, and five washings. The produced essential oil contains >77% of polyphenols. *In vitro* cytotoxicity was examined using an MTT assay against HeLa and Raji cell lines. The essential oil's capability to inhibit the proliferation of HeLa and Raji cell lines was studied under some conditions presenting IC_50_ values of 0.13 and 0.57 *μ*g/mL, respectively. The essential oil was evaluated for its potential as an antioxidant by using *in vitro* models, such as phosphomolybdenum, DPPH, and H_2_O_2_ methods, in comparison with the synthetic antioxidant BHT (butylated hydroxytoluene) and ascorbic acid (vitamin C) as positive controls. The ammonium phosphomolybdate potency in the present study is of the order of 108.75 ± 32.63 mg of essential oil/equivalent to 1 mg of vitamin C in terms of antioxidant power, and the antioxidant activity of DPPH-H_2_O_2_ was 21.3% and 55.2%, respectively. The *Cinnamomum zeylanicum* Blume essential oil (CEO) covers important antioxidant and antiproliferative effects. This can be attributed to the presence of few minor and major phenolic compounds.

## 1. Introduction

Essential oils extracted from aromatic and medicinal plants have been used since antiquity in order to exploit their biological activities [1, 2]. Essential oils have found a large application. Most of them are used as fragrances in perfumery, food additives, beverages, and in the pharmaceutical industry [3]. They have also been employed in folk medicine for thousands of years [4].

In recent times, a growing interest in natural products has been shown due to their availability, fewer side effects, and/or low toxicity as well as better biocomposition compared to the chemical drugs or molecules used in chemotherapy. In fact, medicinal herbs represent a very good alternative to chemical drugs [5–7].

Actually, medicinal plants can be used in the treatment of several diseases [8–11], and a considerable number of essential oils are considered as promising natural compounds, thanks to their different biological activities (e.g., antibacterial, antifungal, antiviral, antioxidant, anticancer, immunomodulatory, analgesic, and anti-inflammatory actions) [12, 13].

Based on their composition, some essential oils have been explored particularly for their antiproliferative properties and cytotoxicity effect against some tumor cell lines [14] and have been interesting for the treatment and prevention of diseases related to inflammation or ROS production [15].

In this perspective, we chose to explore more properties of the cinnamon plant, widely addicted in culinary preparations and aroma condiments in Mediterranean countries. Several parts of this plant, such as the bark, leaves, flowers, fruits, and roots, have medicinal and gastronomic applications. Also, *Cinnamomum zeylanicum* Blume essential oil, yellow-colored oil with a delicate aroma and sweet pungent taste, having a specific gravity of 1.010–1.030 and a solubility in 70% alcohol [16], is amply used in food processing, cosmetics, flavorings, confectionaries, and pharmaceutical industries [17], and also to treat inflammatory diseases [18] and antifungal diseases [19].

The relative volatile components can generally be classified into monoterpenes, sesquiterpenes, and phenylpropenes. The chemical composition of the materials obtained from the different parts of the plant exhibit considerable variation, resulting in different pharmacological effects [20–22].

The first aim of the present work was to optimize the extraction yield of essential oil from *Cinnamomun zeylanicum* Blume using four experimental factors and to examine the corresponding components in order to identify the importance of the studied plant consumption. The second one was to evaluate the antioxidant capacity and antiproliferative effects of the extracted essential oil against human cell lines. This is to define an important application in the medical field.

## 2. Materials and Methods

### 2.1. Essential Oil Extraction

The *Cinnamomum zeylanicum* Blume was purchased from a local market in Sfax, in the south of Tunisia. For each experience, 50 g of *Cinnamomum zeylanicum* Blume sample was cut into small pieces and immerged into distilled water with a ratio of about 9 mL/g (in total, there are 26 experiences designed using response surface methodology; see section 2.2). The hydrodistillation system was used to extract the essential oil of the studied matter. Under the effect of heat, the plant cells burst and release their oil, then the steam of water and oil rise to reach a condensation system, where the cool water circulates with a fixed flow. The hydrodistillation gives a whitish distillate. It is an emulsion of *Cinnamomum zeylanicum* Blume essential oil.

The salting-out separates a product from a reaction mixture. This step consists in saturating the distillate with ionic compounds, sodium chloride (NaCl), to increase the difference in polarity between the aqueous and organic phases, and thus improve their separation. This allows separating the molecules of the essential oil from the water, to pass into the organic solvent during the liquid-liquid extraction step. The salting step is carried out under different saline solution concentrations.

The recovery of the essential oil from salt-saturated distillate is not possible by simple decantation. The liquid-liquid extraction is then used with an organic solvent (cyclohexane) to extract them from the mixture. A few washes (3, 4 or 5) with the used solvent were applied. In the case of five washes (for example), the volumes of 70, 50, 40, 40, and 40 mL were used successively. The solvent is then partially or completely removed by evaporation in a rotary evaporator.

After that, the extract was weighted, and the yield was calculated (equation (1)):

(1) $$\begin{array}{c} Y\left(\text{g}/100\text{ g d}.\text{b}.\right)=\frac{\text{mass of essential oils}}{\text{mass of dried matter}}\times 100. \end{array}$$

The mass of dried matter was determined after 24 h of drying at 105°C.

### 2.2. Optimization of Essential Oil Extraction Yield Using the Response-Surface Methodology

The response-surface methodology (RSM) using central composite design type was investigated to optimize the extraction yield (*Y*) of the essential oil from *Cinnamomum zeylanicum* Blume. Four factors were tested in this work: the water flow used for the condensation in hydrodistillation (noted by *x*_1_); the size of cut samples (*x*_2_); the saline solution concentration used for washing of the extracted essential oil (*x*_3_); and the number of washings of the obtained extract (*x*_4_). The washing was carried out with hexane as a solvent.  Table 1 shows the used levels for each factor.

Table 2 shows the obtained experimental conditions (26 experiences) and the corresponding results presented as the obtained experimental extraction yield (g/100 g d.b.). The experimental conditions are composed of 16 factorial points (the first ones), 8 star-points (from 17^th^ to 24^th^), and two central points (25^th^ and 26^th^). The experimental design was carried out with STATISTICA 12.0 Software, StatSoft, Inc.

The corresponding second-degree model with interactions, presenting the yield (*Y*) as a function of the different factors (*x*_*i*_) is shown by equation (2):

(2) $$\begin{array}{c} Y=\beta_{0}+\overset{n}{\underset{i=1}{\sum }}\beta_{i}\cdot x_{i}+\overset{n-1}{\underset{i=1}{\sum }}\overset{n}{\underset{j>i}{\sum }}\beta_{ij}\cdot x_{i}\cdot x_{j}+\overset{n}{\underset{i=1}{\sum }}\beta_{ii}\cdot x_{i}^{2}+\varepsilon , \end{array}$$

where *β*_0_, *β*_*i*_, *β*_*ij*_, and *β*_*ii*_ are the regression coefficients, *ε* is the residual value between calculated and experimental data, *x*_*i*_ is the centered reduced level of factor “*i*” (value is equal to −1, 0 or +1), and *n* is the factors number.

The coefficients determination was obtained using least squares method.

### 2.3. GC/MS Analysis Conditions

An Agilent-Technologies Model 6890N Network Gas Chromatograph system was used to analyze the essential oil. It is equipped with a flame ionization detector and HP-5MS capillary column (30 m × 0.25 mm × 0.25 *μ*m; Agilent-Technologies, Little Falls, CA, USA). The temperatures of the injector and detector were set at 250 and 280°C, respectively. The column temperature was planned from 35 to 250°C with a rate of 5°C/min, with the lower and upper temperatures being held for 3 and 10 min, respectively. The flow rate of helium (used as the carrier gas) was 1.0 mL/min. A sample of 1.0 *μ*L was injected with split mode (split ratio, 1 : 100). All quantifications were approved using a built-in data handling program provided by the manufacturer of the gas chromatograph. The composition was carried out as a relative percentage of the total peak area. The essential oil constituent's identification was done on the retention times comparison to n-alkanes, related to published data and spectra of authentic compounds. Therefore, compounds were identified and authenticated using their mass spectra compared to the Wiley version 7.0 library.

### 2.4. Cell Culture Lines

The continuous human cell lines HeLa (epithelial cervical cancer cell line) and Raji (human Burkitt's lymphoma-derived cell line) were examined for the cytotoxicity effect of plant extract. Those cell lines were grown in RPMI 1640 medium (Gibco) supplemented with 10% (v/v) of foetal calf serum (FCS) and 2 mM of L-glutamine in tissue culture flasks (Nunc). They were passed twice a week and kept at 37°C in a humidified atmosphere of 95% air and 5% CO_2_.

### 2.5. MTT Cell Proliferation Assay

MTT (3-(4,5-dimethylthiazol-2-yl)-2,5-diphenyltetrazolium bromide cell proliferation assay measures the cell proliferation rate and, conversely the reduction in cell viability when metabolic events lead to apoptosis or necrosis. The yellow compound MTT (Sigma) is reduced by mitochondrial dehydrogenases to the water insoluble blue formazan compound, depending on the cell viability.

Cells were grown on microtiter plates (200 *μ*L of cell suspension/well) in 96 well microplates with serial dilutions of extract. After 72 h, 20 *μ*L of MTT solution (5 mg/mL) was added in each well. The plate was incubated during 4 h at 37°C in a CO_2_ incubator. After that, 180 *μ*L of medium was removed from each well, and 180 *μ*L of DMSO:methanol (50 : 50) was added to each sample. The preparations were mixed thoroughly on a plate shaker with each cell containing formazan crystals. When all the crystals were dissolved, absorbance was measured at 570 nm with a microplate reader (Elx 800 microplate reader).

### 2.6. Cytotoxicity Effect of *Cinnamomum zeylanicum* Blume Essential Oil

To test the cytotoxic effect of *Cinnamomum zeylanicum* Blume essential oil on studied human cell lines, cells were treated with various concentrations of essential oil ranging from 0.125 to 4 *μ*g/mL for HeLa and from 12 to 0.375 *μ*g/mL for Raji, for 48 h, and then submitted to the MTT test.

### 2.7. Antioxidant Capacity Assays

#### 2.7.1. Phosphomolybdenum Assay

Essential oil samples (100 *μ*L) were mixed with 1 mL of the phosphomolybdenum reagent (600 mM sulfuric acid, 28 mM sodium phosphate, 4 mM ammonium molybdate) [23]. Then, the mixture was incubated at 95°C for 90 min and cooled to room temperature. Subsequently the absorbance was measured at 695 nm. In order to estimate the percentage of molybdenum reduced by the tested essential oil, a standard curve was constructed using ascorbic acid. EC_50_ (mg/mL) corresponds to the effective concentration at which the total antioxidant activity (TAA) at 50% was obtained by interpolation from linear regression analysis. As a positive control, ascorbic acid was used. The values are presented as the means of the triplicate assay.

#### 2.7.2. 2,2-Diphenyl-1-Picrylhydrazyl (DPPH) Free Radical Scavenging Activity Assay

The antioxidant activity of essential oil was evaluated by examining its ability in quenching the stable-free radical DPPH. The radical scavenging activity of essential oil against DPPH-free radicals was measured using the method of [24]. 750 *μ*L of essential oil with five decreasing solutions concentrations was prepared (1000, 500, 250, 125, and 62.5 *μ*g/mL), which was added to 1500 *μ*L of DPPH solution (100 *μ*M). The mixture was allowed to reach a steady state at room temperature for 30 min. The staining of DPPH was determined by measure the absorbance at 517 nm with a spectrophotometer. All determination was approved out in triplicate. Synthetic antioxidant reagent BHT (Butylated Hydroxy-Toluene) was used as a positive control. Negative control used methanol solvent. The DPPH radical scavenging activity was calculated according to the following equation (3):

(3) $$\begin{array}{c} I\left(\%\right)=\frac{\text{Abs total DPPH}-\text{Abs sample}}{\text{Abs total DPPH}}\times 100. \end{array}$$

#### 2.7.3. Hydrogen Peroxide Radical Scavenging Assay (H_2_O_2_)

For hydrogen peroxide scavenging activity of the essential oil, it was determined using the method described by [25]. 2 mL of plant extract, vitamin C, or gallic acid was prepared in ethanol at different concentrations (1–0.0625 mg/mL) and mixed with 1.2 mL of 40 mM hydrogen peroxide (H_2_O_2_) solution prepared in phosphate buffer (0.1 M, pH 7.4). The reaction mixture was incubated for 40 min. The absorbance of the solution was then measured at 230 nm. The H_2_O_2_ scavenging percentage was calculated according to equation (4):

(4) $$\begin{array}{c} I\left(\%\right)=\frac{A_{230}\text{ of blank}-A_{230}\text{ of sample}}{A_{230}\text{ of blank}}\times 100. \end{array}$$

### 2.8. Statistical Analyses

The experimental values were presented as the means ± standard deviation (SD). The one-way analysis of variance (ANOVA) test was used to determine the significant and insignificant factors/interactions in the design of the experiments part. The adopted significance level in this work is 95% (corresponding to a probability value *p* < 0.05).

All statistical analyses were carried out with STATISTICA 12.0 Software, StatSoft, Inc.

## 3. Results and Discussion

### 3.1. Optimization of Essential Oil Yield

The experimental results of essential oil yield obtained via the response-surface methodology (RSM) are presented in Table 2. The obtained yields were considered as very important compared to the previous works [26–28] and in the same order with those obtained by [29]. The identified model using the method of least squares is presented as in equation (5):

(5) $$\begin{array}{c} \hat{Y}=\;2.18-0.04\cdot x_{1}+0.06\cdot x_{2}-0.03\cdot x_{3}+0.06\cdot x_{4}+0.09\cdot x_{1}^{2}-0.40\cdot x_{2}^{2}-0.35\cdot x_{3}^{2}+0.29\cdot x_{4}^{2}-0.03\cdot x_{1}\cdot x_{2}-0.04\cdot x_{1}\cdot x_{3}+0.04\cdot x_{1}\cdot x_{4}+0.19\cdot x_{2}\cdot x_{3}+0.02\cdot x_{2}\cdot x_{4}+0.02\cdot x_{3}\cdot x_{4}. \end{array}$$

The statistical test of ANOVA (Table 3) shows that only three factors (from four in total) influence significantly the essential oil yields (*p* < 0.05): sample size, the concentration of the saline solution, and the number of washings. In fact, these influences are only in quadratic form and are very high. The order influence importance is as follows: sample size (*p*=0.007), saline solution concentration (*p*=0.014), and number of washings (*p*=0.032). Moreover, the only interaction having a significant effect on the response is that between the sample size and saline solution concentration. It is considered like the most important influence, since the corresponding probability is very low (*p*=0.002). All these influences show that the mass transfer during the extraction of the essential oil is favored by some particular conditions defining the optimum of extraction which will be determined after the model validation. This behavior was observed previously in essential oil extraction from lemongrass (*Cymbopogon flexuosus*) [10], from *Carex meyeriana* [30], and from *Lawsonia inermis* [11] using hydrodistillation.

**Figure 1:**
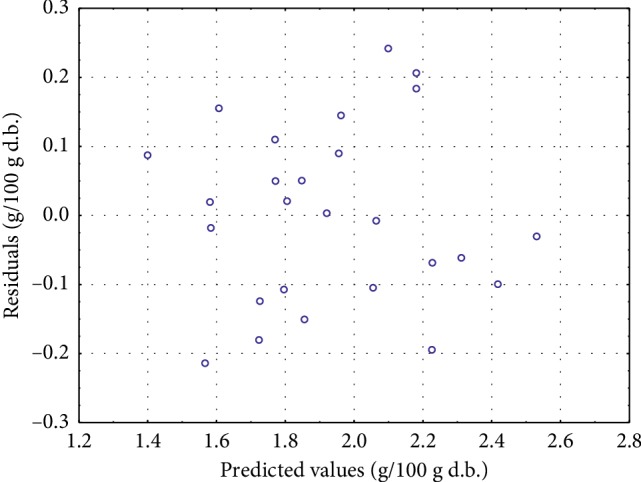
Residual values as function of predicted values.

The proposed model shows a highly significant regression (*p*=0.014), a high coefficient of determination (*R*^2^ = 83.37%), and an interesting adjusted coefficient of determination (*R*_A_^2^=62.20%). It shows also a very low-root-mean-square error (RMSE = 0.193). This value, compared to the extraction yield range, shows the adequacy of the model. In addition, the residual values (*ε*) presented as a function of fitted values show a random profile and very low values (Figure 1). Moreover, Table 3 also presents the lack of fit variance (0.041) which is nonsignificant (*p* > 0.05) compared to the pure error variance (0.0002). This last criterion shows a very low value, confirming the repeatability of the experience. All these criteria make the proposed model very adapted and valid for all experimental ranges chosen for this work.

**Figure 2:**
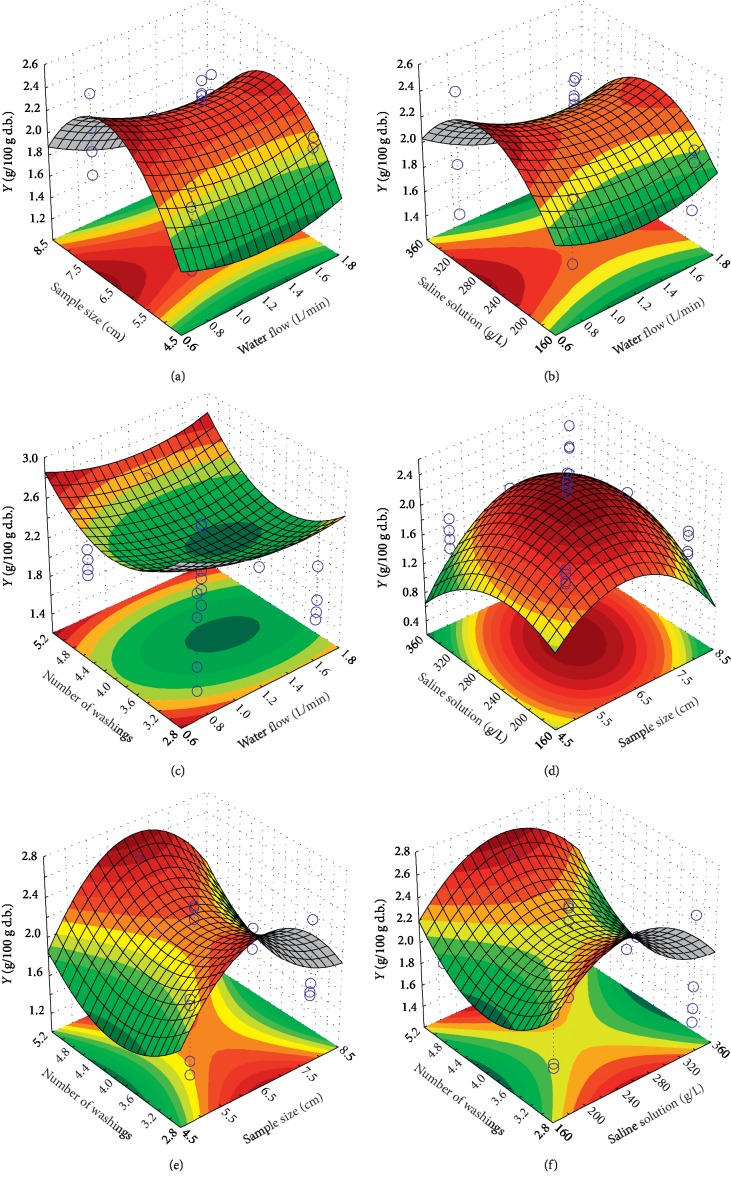
Yields of essential oil as function of (a) water flow and sample size; (b) water flow and saline solution concentration; (c) water flow and number of washings; (d) sample size and saline solution concentration; (e) sample size and number of washings; and (f) saline solution concentration and number of washings.

Figure 2 presents the variation of the yield of essentials oil as a function of the four different factors. The significant quadratic influences of all factors (except the first one: water flow) are clearly shown in Figure 2.  Figure 2(d) displays also the existing interaction between the size of the samples and the saline solution concentration. In addition, the importance of the factors' influences is also noted in all subfigures of Figure 2. For example, Figure 2(e) exhibits the importance of the sample size influence (*x*_2_) compared to the number of washings influence (*x*_4_). All these results are determined previously by ANOVA. Generally, this response behavior gives us a maximum that can be obligatorily identified in the chosen factors' experimental range. This is considered very advantageous for our work.

**Figure 3:**
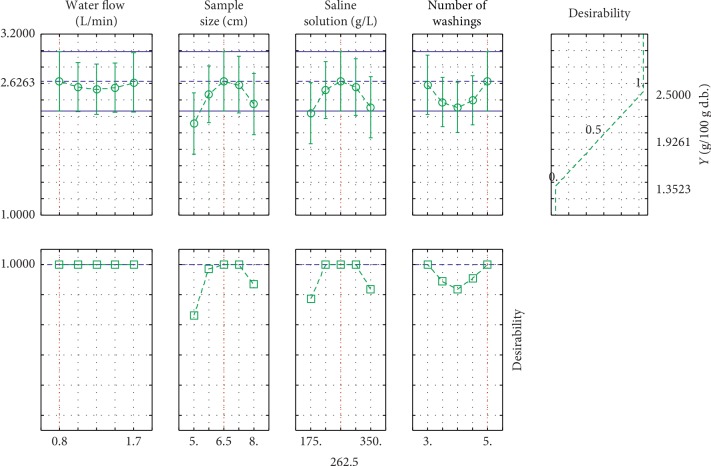
Profiles for predicted values and desirability.

The identified optimum is *Y* = 2.6 g/100 g d.b. with desirability of 100%. This value is determined by STATISTICA Software. The optimum is determined using a minimum water flow of 0.8 mL/min, a sample size of 6.5 cm, a saline solution concentration of 262.5 g/L, and maximum times of washing (5 times) (Figure 3).

### 3.2. Chemical Composition of Essential Oil of *Cinnamomum zeylanicum* Blume

Analysis of the chemical composition of *Cinnamomum zeylanicum* Blume essential oil with GC-MS showed 23 compounds which represent 99.39% of total oil (Table 4). The major groups of constituents were cinnamaldehyde (77.34%), *trans-*cinnamyl acetate (4.98%), benzene dicarboxylic acid (3.55%), *α*-pinene (2.6%), and coumaric acid (1.79%) (Table 4).

The identified components are highly researched in the case of chemotherapy by *Cinnamomum zeylanicum* Blume essential oil. The results are in accord with those cited by [31] for different essential oils extracted from different vegetal sources.

### 3.3. Cytotoxicity Assays

**Figure 4:**
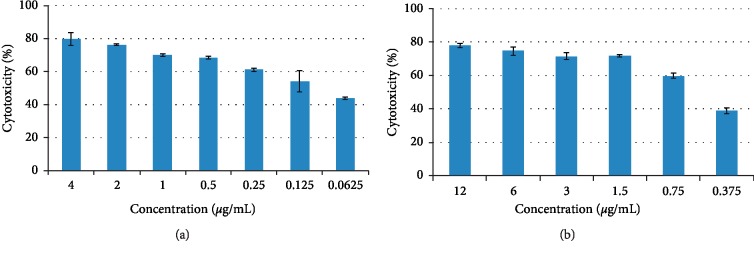
Cytotoxic activity of *Cinnamomum zeylanicum Blume* essential oil determined by the MTT assay: (a) HeLa and (b) Raji.

Many studies have shown a considerable cytotoxic activity of the essential oils, usually without being mutagenic, in various organisms [12, 32]. Therefore, these essential oils seem to be useful as potential antitumor agents [27]. In this context, the effect of the different concentrations of *Cinnamomum zeylanicum* Blume essential oil against HeLa (0.0625 to 4 *μ*g/mL) and Raji (0.375 to 12 *μ*g/mL) cell lines was studied in this work (Figure 4).

For both lines, an increase of the cytotoxicity with the essential oil's concentration was observed indicating a dose-dependent effect. An examination of the obtained results showed that the cytotoxic effects of *Cinnamomum zeylanicum* Blume essential oil were different between HeLa and Raji cell lines.

The IC_50_ of *Cinnamomum zeylanicum* Blume essential oil (the required tested oil concentration to reduce the cell survival fraction to 50% of the control) was obtained with 0.13 *μ*g/mL against HeLa, when IC_50_ was obtained with 0.57 *μ*g/mL against the Raji cell line.

As a result, we noted that all concentrations of the *Cinnamomum zeylanicum* Blume essential oil showed important regressing viability of the HeLa cell line compared to the Raji cell line. For a 0.25 *μ*g/mL of the essential oil, we noted 60 % of destructed HeLa cells against 0.75 *μ*g/mL to destruct the same percentage of Raji cells. Therefore, 4 *μ*g/mL of the studied essential oil was able to destroy 79.7% of HeLa cells. Using concentration higher than 4 *μ*g/mL and more than 12 *μ*g/mL, all HeLa and Raji cells were destructed, respectively.

These differences could be attributed to a diversity of molecules of the essential oil and affinity effect on one type of tumor cell more than on others. According to the American National Central Institute, plant extracts that show cytotoxicity with an IC_50_ < 30 *μ*g/mL can be considered as potential agents for the development of anticancer drugs [33]. Then, the optimized essential oil of cinnamon extracted in this work could be considered as a promote drug against HeLa and Raji cell lines.

Besides, Unlu et al. [27] approved the cytotoxic and apoptotic effects of this essential oil on ras active 5RP7 and normal fibroblasts (F2408). With IC_50_ values less than 20 *μ*g/mL, the 5RP7 cells were affected stronger than the normal cells (F2408). Also, they mentioned the induction apoptosis mechanism of 5RP7 cells at the high level of this oil.

Recently, Pistelli et al. [34] showed a distinguished *Cinnamomum zeylanicum* essential oil effect against two tested breast cancer cell lines with an IC_50_ value of around 20 ppm against MCF7, MDA-MB-231, and one malignant tumor cell (SH-SY5Y) and less effective than 6 ppm on human chronic myelogenous erythroleukemia (K562) and 56.1 ppm on the T47D tumor cell line.

Bouyahya et al. [35] presumed that many molecules in essential oils have antitumor property and cytotoxic activity against human tumors cell lines, especially carvacrol, thymol, and eugenol groups of alcohol as linalool and groups of aldehydes as cinnamic aldehyde. This different potential of the cytotoxicity is the resultant of essential oil molecules with varying degrees of proven cell death effect. The obtained results can be related to the composition of the essential oil extracted from the studied matter (*Cinnamomum zeylanicum* Blume containing more than 77% of cinnamaldehyde). In fact, according to the literature, the cytotoxicity effect of the essential oil against tumor cell lines could be due to the presence of some monoterpenes and sesquiterpenes and also to the presence of the highest content of polyphenols (>77%) which certainly gives a very important antioxidant property for this essential oil [36–38].

Leela [39] has reported that the cinnamicaldehyde and the hydrocinnamic acid from *Cinnamomum zeylanicum* Blume essential oil were able to react as trappers of the free peroxide radicals and prevent oxidative damages. Consequently, some reports defend the relationship between antioxidant activity and cytotoxicity effect [40] and maintain that antioxidant activity of essential oil of *Cinnamomum zeylanicum* Blume can contribute to its cytotoxic activity [40, 41].

### 3.4. Antioxidant Capacity Assays

The antioxidant activity of some cinnamon species essential oil and its constituents has been extensively investigated in different systems [42–46].

**Figure 5:**
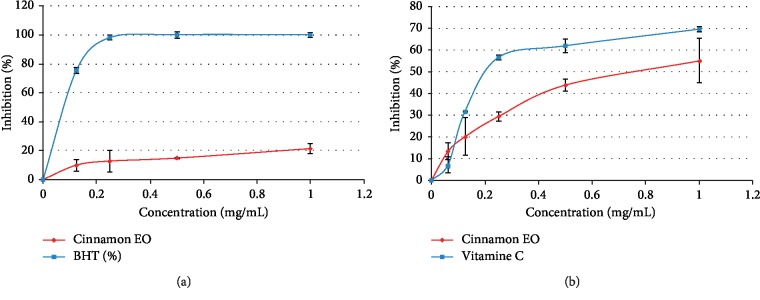
The scavenging capacity of (a) DPPH and (b) H_2_O_2_.

Then, in our case the ammonium phosphomolybdate potency of *Cinnamomum zeylanicum* Blume essential oil was of the order of 108.75 ± 32.63 mg of essential oil/equivalent to 1 mg of vitamin C in terms of antioxidant power. Also, it exhibited satisfactory antioxidant power to scavenging DPPH (Figure 5(a)) and H_2_O_2_ (Figure 5(b)) free radical compared to their synthetic antioxidant (BHT; vitamin C) positive control. The essential oil of cinnamon was capable of scavenging hydrogen peroxide in an amount dependent manner. 0.7 mg/mL of essential oil of cinnamon exhibited 50% scavenging activity on hydrogen peroxide. Recently, Abeysekera et al. [47] showed that antioxidant and also glycemic regulatory properties were related to different maturity stages of authenticates leaves (not a bark) of *Ceylon cinnamon*.

It seems that the antioxidant activity of cinnamon oil is due to the presence of some monoterpenes such as *α*-pinene, a known potent antioxidant [48] and several sesquiterpenes [49], and it is assumed that the contribution of minor and major compounds exhibited this activity which can be attributed to one or few active molecules [48].

## 4. Conclusion

The present study has reported an optimum extraction yield of *Cinnamomum zeylanicum* Blume essential oil (CEO) using RMS with its composition (principally cinnamaldehyde 77.34% and *trans-*cinnamyl acetate 4.98%). This oil has shown an important cytotoxic effect against HeLa and Raji cell lines and considerable antioxidant activity. CEO could be used as a potential and beneficial drug in phytotherapy disease treatment.

Further researches are needed to explore the composition-effect-mechanism-dose relations using more *in vitro*/*in vivo* bioassay tests.

## Figures and Tables

**Table 1:** Used levels for the four factors.

Factor	Coded factor	Level		
		Low (−1)	Center (0)	High (+1)
Water flow (L/min)	*x*_1_	0.80	1.25	1.70
Sample size (cm)	*x*_2_	5.0	6.5	8.0
Concentration of saline solution (g/L)	*x*_3_	175	262.5	350
Number of washings	*x*_4_	3	4	5

**Table 2:** Experimental conditions and obtained result.

Essay	*x*_1_	*x*_2_	*x*_3_	*x*_4_	*Y* (g/100 g d.b.)
1	−1	−1	−1	−1	2.22
3	+1	−1	−1	−1	2.16
9	−1	+1	−1	−1	1.69
11	+1	+1	−1	−1	1.65
2	−1	−1	+1	−1	1.43
4	+1	−1	+1	−1	1.57
10	−1	+1	+1	−1	2.47
12	+1	+1	+1	−1	1.78
5	−1	−1	−1	+1	2.03
7	+1	−1	−1	+1	2.06
13	−1	+1	−1	+1	1.92
15	+1	+1	−1	+1	1.98
6	−1	−1	+1	+1	1.86
8	+1	−1	+1	+1	1.69
14	−1	+1	+1	+1	2.14
16	+1	+1	+1	+1	2.17
17	−1	0	0	0	2.37
18	+1	0	0	0	2.28
21	0	−1	0	0	1.63
22	0	+1	0	0	2.00
23	0	0	−1	0	1.80
24	0	0	+1	0	1.93
19	0	0	0	−1	2.45
20	0	0	0	+1	2.64
25	0	0	0	0	2.49
26	0	0	0	0	2.52

**Table 3:** ANOVA table.

Source	SS	df	MS	F	*p*
Regression	2.051	14	0.147	3.940	0.014
*x*_1_	0.032	1	0.032	0.856	0.375
*x*_1_^2^	0.020	1	0.020	0.547	0.475
*x*_2_	0.069	1	0.069	1.860	0.200
*x*_2_^2^	0.399	1	0.399	10.739	0.007
*x*_3_	0.011	1	0.011	0.309	0.590
*x*_3_^2^	0.315	1	0.315	8.464	0.014
*x*_4_	0.057	1	0.057	1.541	0.240
*x*_4_^2^	0.221	1	0.221	5.939	0.033
*x*_1_ · *x*_2_	0.018	1	0.018	0.496	0.496
*x*_1_ · *x*_3_	0.026	1	0.026	0.695	0.422
*x*_1_ · *x*_4_	0.020	1	0.020	0.538	0.479
*x*_2_ · *x*_3_	0.591	1	0.591	15.888	0.002
*x*_2_ · *x*_4_	0.008	1	0.008	0.203	0.661
*x*_3_ · *x*_4_	0.007	1	0.007	0.183	0.678
Residual error	0.409	11	0.037		
Lack of fit	0.4088	10	0.041	158.327	0.062
Pure error	0.0002	1	0.0002		
Total	2.460	25			

SS: sum of squares; df: degree of freedom; MS: mean square; F: Fisher report; *p*: *p* value.

**Table 4:** Chemical composition, retention time, and percentage composition of the *Cinnamomum zeylanicum* Blume essential oil.

N°	Compound	Brute formula	Retention time (min)	Percentage (%)
1	Benzaldehyde	C_7_H_6_O	10.49	0.23
2	1,8-Cineole	C_10_H_18_O	14.85	**3.19**
3	*γ*-Terpinene	C_10_H_16_	16.60	0.16
4	Linalool	C_10_H_18_O	19.65	0.30
5	Camphenilol	C_9_H_16_O	22.67	0.02
6	Borneol	C_10_H_18_O	24.06	0.31
7	Cyclohexene	C_6_H_10_	24.94	0.74
8	*α*-Pinene	C_10_H_16_	25.96	**2.60**
9	*α*-Terpinene	C_10_H_16_	27.83	0.38
10	Cinnamaldehyde	C_9_H_8_O	33.53	**77.34**
11	*trans*-Caryophyllene	**C_15_H_24_**	41.94	0.13
12	Eugenol	C_10_H_12_O_2_	43.09	0.02
13	Hydrocinnamic acid-2,3-13C2	C_9_H_10_O_2_	43.67	0.13
14	*trans*-Cinnamyl acetate	C_11_H_12_O_2_	45.61	**4.98**
15	Coumaric acid	C_9_H_8_O_3_	46.24	**1.79**
16	Propenoic acid	C_3_H_4_O_2_	46.89	0.75
17	*δ*-Cadinene	**C_15_H_24_**	48.18	0.14
18	Caryophyllene oxide	C_15_H_24_O	51.80	0.17
19	Naphthalenol	C_10_H_8_O	54.07	0.05
20	Hexadecanoic-d31	C_16_H_32_O_2_	71.63	0.37
21	9-Octadecenoic acid	C_18_H_34_O_2_	76.81	**1.32**
22	Phthalic acid	C_6_H_4_(COOH)_2_	95.28	0.72
23	1,4-Benzenedicarboxylic acid	C_16_H_22_O_4_	95.28	**3.55**
				Total (%) = **99.39**

## Data Availability

No data were used to support this study.

## Abbreviation

df: Degree of freedom (dimensionless)
F: Fisher report (dimensionless)
MS: Mean square or variance
*p*: Value of probability (dimensionless)
*R*^2^: Coefficient of determination (dimensionless)
*R*_A_^2^: Adjusted coefficient of determination (dimensionless)
RMSE: Root-mean-square error
SS: Sum of squares
*x*_*i*_: Reduced centered variable of the *i*^th^ factor (dimensionless)
*Y*: Yield of essential oil extraction provided in g/100 g dry basis (g/100 g d.b.).
